# Filtering the Junk: Assigning Function to the Mosquito Non-Coding Genome

**DOI:** 10.3390/insects12020186

**Published:** 2021-02-22

**Authors:** Elise J. Farley, Heather Eggleston, Michelle M. Riehle

**Affiliations:** Department of Microbiology and Immunology, Medical College of Wisconsin, Milwaukee, WI 53226, USA; efarley@mcw.edu (E.J.F.); heggleston@mcw.edu (H.E.)

**Keywords:** miRNA, lncRNA, enhancer, non-coding, regulatory element

## Abstract

**Simple Summary:**

In eukaryotes, the fraction of the genome not coding for proteins vastly outsizes the portion containing protein-coding genes. This non-coding genome, once termed “junk”, was thought for decades to be inconsequential to the biology of an organism. It is now widely acknowledged that elements within the non-coding genome serve important gene-regulatory functions impacting when, where, and to what levels genes and their protein products are expressed. Without an amino acid-like code to decipher non-coding regulatory elements within the genome, significant technology development has aided in their discovery. Currently, genome-wide identification of non-coding regulatory elements is an active area of research with significant progress made in humans, mice, and other model organisms. However, work to address the roles of these elements in mosquito disease vectors is in its infancy. In this article, we review existing methodology to generate genome-wide catalogs for three classes of non-coding elements and discuss their use in mosquito disease vectors and other insects.

**Abstract:**

The portion of the mosquito genome that does not code for proteins contains regulatory elements that likely underlie variation for important phenotypes including resistance and susceptibility to infection with arboviruses and Apicomplexan parasites. Filtering the non-coding genome to uncover these functional elements is an expanding area of research, though identification of non-coding regulatory elements is challenging due to the lack of an amino acid-like code for the non-coding genome and a lack of sequence conservation across species. This review focuses on three types of non-coding regulatory elements: (1) microRNAs (miRNAs), (2) long non-coding RNAs (lncRNAs), and (3) enhancers, and summarizes current advances in technical and analytical approaches for measurement of each of these elements on a genome-wide scale. The review also summarizes and highlights novel findings following application of these techniques in mosquito-borne disease research. Looking beyond the protein-coding genome is essential for understanding the complexities that underlie differential gene expression in response to arboviral or parasite infection in mosquito disease vectors. A comprehensive understanding of the regulation of gene and protein expression will inform transgenic and other vector control methods rooted in naturally segregating genetic variation.

## 1. Introduction

Phenotypic diversity in mosquitoes cannot be explained using only variability among protein-coding regions of the genome. Rather, phenotypic variation may be the result of differences in gene and protein expression driven by changes in three-dimensional chromatin structure and regulatory elements residing within the non-coding, “junk”, regions of the genome. In 1972, geneticist Susumu Ohno coined the term “junk DNA” to describe all non-coding portions of the genome. These “junk” regions, comprising up to 80% of the genome, are scattered randomly throughout the genome and often arise from transposition, or movement of DNA from one part of the genome to another [1]. The composition of the DNA sequence in these non-coding regions is often low-complexity and enriched for repeated sequences, which can make functional characterization of these areas difficult. In the nearly 50 years since the term “junk DNA” was coined, the notion that non-coding DNA is “junk” has been replaced by information confirming non-coding DNA not only has a function, but exerts dynamic control over coding region gene expression.

Despite the realization that the non-coding part of the genome has function, neither identification of non-coding regulatory elements nor assigning function to genetic variation in these non-coding portions of the genome are yet routine in any organism and certainly not for mosquitoes. Unlike protein-coding sequences, where an amino acid code allows delineation of an open reading frame and the ability to interpret synonymous and nonsynonymous substitutions, no such code exists to either identify non-coding elements nor interpret the functional consequence of genetic variation within them. These non-coding elements include microRNAs (miRNAs), long non-coding RNAs (lncRNAs), and enhancers, a type of cis-regulatory element (CRE), among others. Of the significant single nucleotide polymorphisms (SNPs) associated with phenotypes in human genome-wide association studies (GWAS), only 5–10% are protein-coding variants, and >90% of GWAS hits are non-coding SNPs [2,3,4]. In a recent association mapping study of *Anopheles* desiccation resistance, the most significant SNPs were located in non-coding regions [5]. Non-coding regulatory elements represent a sort of “dark genome” that appears to be responsible for the vast majority of phenotypic variation in animals but is currently beyond our ability to identify and easily interpret. Prior to understanding the impacts of non-coding genetic variation on phenotype, comprehensive identification of non-coding regulatory elements is necessary. Such activities have become more common in prominent mosquito disease vectors.

In addition to enhancers, miRNAs, and lncRNAs, siRNAs (small interfering RNAs) and piRNAs (PIWI-interacting RNAs) can also play important roles in modulating gene expression. siRNAs are double-stranded non-coding RNA molecules, 20–27 base pairs in length, that operate within the RNA interference pathway. siRNAs interfere with the expression of genes with complementary sequence by degrading mRNA following transcription, thereby preventing translation. piRNAs are a large class of small non-coding RNA molecules 21–35 nucleotides in length that form RNA-protein complexes through interactions with Argonaute proteins and silence transposable elements, regulate gene expression, and fight viral infection. piRNA complexes are also involved in epigenetic gene regulation. In-depth reviews of the roles of both siRNA and piRNA in insects have been published recently [6,7,8].

**Figure 1:**
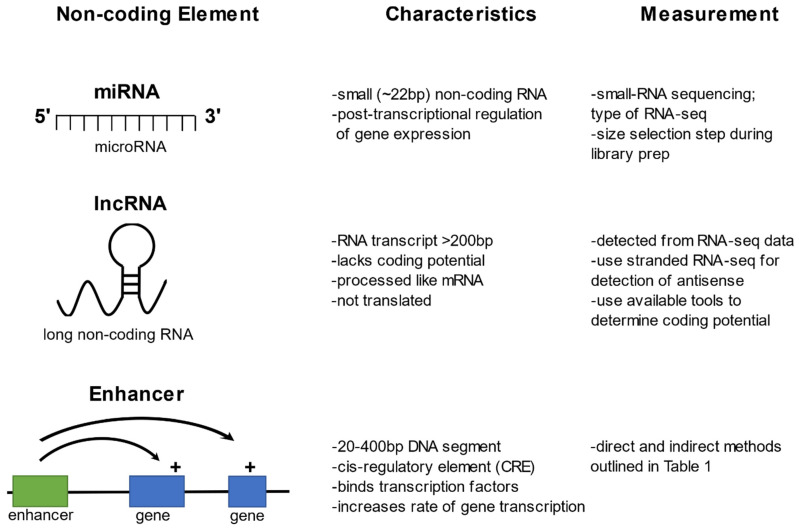
Three regulatory elements located in the non-coding portion of the genome.

This review focuses primarily on three non-coding regulatory elements; miRNAs, lncRNAs, and transcriptional enhancers (Figure 1), and discusses relevant technological advances and analytical approaches for genome-wide detection of these non-coding elements. Technological limitations, as well as potential areas of experimental bias, are discussed, as are impacts of genetic variation within these non-coding elements. Implementation of these methods within mosquito disease vectors and relevant results, particularly as they pertain to arboviral or parasitic disease transmission, are also discussed.

## 2. MicroRNAs

MicroRNAs (miRNAs) are non-coding RNA molecules 18–24 nucleotides in length that regulate gene expression post-transcriptionally [9]. miRNAs are present in a wide variety of organisms, including plants, vertebrates, insects, and some viruses (for reviews see [10,11,12,13]). At any one time, an organism expresses hundreds of miRNAs that bind by sequence complementarity to target messenger RNA (mRNA) [14,15]. miRNAs mediate mRNA repression by binding to an Argonaute protein and forming a miRNA-induced silencing complex (RISC) where they guide the complex to target mRNAs (see reviews for more details [16,17,18]). Genetic variation within miRNAs may affect their binding efficiency to target genes and in turn modulate gene expression. The effects of SNPs in miRNAs are just beginning to be investigated, but a handful of disease and phenotypic associations have been detected [19,20]. In mosquitoes, miRNAs have been cataloged, with some functional studies, but the effect of miRNA genetic variation has not yet been examined [12,21,22,23,24].

Techniques for cataloging miRNAs on a genome-wide scale include microarray-based approaches and small RNA sequencing (sRNA-seq). miRNA microarray profiling is a hybridization probe-based system where miRNAs bind to fluorescent probes containing complementary sequence. This experimental approach cannot measure recently annotated or novel miRNAs, and the low signal-to-noise ratio limits the feasibility to detect lowly abundant miRNA [25]. Due to these technological limitations, the majority of recent studies utilize a next-generation sequencing sRNA-seq approach. sRNA-seq allows for the prediction of novel miRNA as sequence data is mapped directly to the genome [26] and does not rely on existing miRNA catalogs. sRNA-seq also allows for the detection of low-abundance miRNA transcripts and yields data on novel miRNA nucleotide sequence [27]. sRNA-seq differs from standard mRNA sequencing by the addition of a size selection step during the library preparation where the small RNAs are isolated from larger RNA molecules through a gel electrophoresis step [28,29]. There are inherent biases of the sRNA-seq technique including the effects that GC content and adaptor/barcode sequences can have on the efficiency of cDNA synthesis prior to sequencing (see recent reviews for more details [30,31]). Sequence reads from sRNA-seq are analyzed using bioinformatic tools to predict novel miRNAs and their functions, miRNA structure, phenotypic association and regulatory targets (see recent reviews [27,32]).

The functions of miRNAs in mosquito species are diverse, including regulation of immune response to pathogens and transcriptional regulation during specific life stages or in different tissues. As many mosquito species have the ability to vector human pathogens such as the arboviruses; dengue, Zika, and chikungunya, as well as *Plasmodium* parasites, understanding the role of miRNAs during the immune responses to these pathogens can be vital in developing novel vector control strategies (see recent reviews [22,33]). A recent study examined the miRNA expression in midguts of *Anopheles anthropophagus* fed either on non-infected or *Plasmodium*-infected blood [29]. In the non-infected blood experiment, nine significantly upregulated and 10 significantly downregulated miRNAs were identified, with one (miR-92a) previously reported as induced upon blood feeding in *Aedes aegypti* [29,34]. Feeding on *Plasmodium*-infected blood elicited up- and downregulation of an additional 13 and 11 miRNAs, some of which have been identified upon *Plasmodium* or *Wolbachia* infection in other mosquito species [29,35,36]. Recent studies have highlighted the complex involvement of miRNAs in the viral response in multiple mosquito vector species with some exhibiting potential proviral effects [37,38] and others potential antiviral effects [21,38,39,40]. A purely bioinformatic approach has been used to identify potential binding sites of *Ae. aegypti* miRNA in the chikungunya, dengue, and Zika viral genomes (no experimental validation was attempted [41]). A study of *Ae. aegypti* miRNA responses to Ross River virus (RRV) infection examined the antiviral response in the fat body and midgut tissues post-inoculation [42] and identified 14 differentially-regulated miRNAs with the majority of differentially expression in fat body at 2 days post inoculation. Prediction of mRNA targets for these miRNAs implicated several genes related to immune response; however, further work is needed to characterize the role of these miRNAs in viral replication [42].

Numerous recent studies have dissected the role of individual miRNAs in mosquito vector competence, mosquito physiology, and insecticide resistance. Work in *Anopheles coluzzii* showed that blood meal-induced miRNA-276 is integral to the regulation of the mosquito reproductive cycle with silencing of miRNA-276 resulting in increased female fertility and decreased *Plasmodium* transmission [43]. In *Anopheles gambiae,* coordinated changes in miRNA expression levels in energy-storing tissues appear to play a role in blood meal-induced metabolic changes observed following feeding [44]. Recent work in *Culex pipiens* implicated miRNAs in differential susceptibility to deltamethrin insecticides and adult reproductive diapause through their impact on ovarian development and lipid abundance [45,46]. A recent review of miRNA expression and function in mosquitoes summarizes further the roles of individual miRNAs in mosquito biology [12]. Taken together, all of these studies emphasize the important regulatory roles miRNAs play in all aspects of mosquito physiology, vector competence, and insecticide resistance. Further, given their importance in vector competence and insecticide resistance, miRNAs are likely to influence vector control methods currently centered on the use of insecticides.

One remaining challenge in the study of miRNAs is characterizing their functional interaction(s) with the mRNAs they regulate. Recent application of the covalent ligation of endogenous Argonaute-bound RNAs (CLEAR)-crosslinking and immunoprecipitation (CLIP) technique [46] in *An. gambiae* has begun to explore physical interactions between miRNAs and their target mRNAs [47,48]. The technique results in the simultaneous capture of thousands of miRNA–mRNA target pairs after direct ligation of the miRNA and its cognate target transcript in endogenous Argonaute–miRNA–mRNA complexes. This recent work not only confirmed known interactions between miR-309 and homeobox gene *SIX4*, but also highlighted many additional interactions for this single miRNA [49]. CLEAR-CLIP assays identified a total of 220 miR-309–mRNA interactions involving 204 distinct mRNA transcripts. CLEAR-CLIP-like approaches are necessary to assign mechanistic function to miRNAs and to specifically identify the mosquito mRNAs they regulate to modulate the whole mosquito phenotype. Knowledge of these interactions will shed light on how genetic variation in either miRNAs or their target mRNAs impacts gene expression.

## 3. Long Non-Coding RNAs

One of the lesser-studied non-coding elements are long non-coding RNAs (lncRNAs), defined as transcripts longer than 200 nucleotides that lack amino acid coding potential. lncRNAs have many mRNA-like characteristics, including that they are transcribed by RNA polymerase II, 5’ capped, polyadenylated, and often spliced [50]. lncRNAs can be sense overlapping, sense intronic, antisense, or they can be intergenic (intergenic lncRNAs are often called lincRNAs for long intergenic non-coding RNA). Based on data showing abundant expression in only certain cells or tissues, lncRNAs are thought to be more tightly regulated than mRNAs [51,52]. lncRNAs have been linked to various biological functions including both cis and trans regulation of gene expression, development, dosage compensation, and imprinting (see recent reviews [53,54,55]). lncRNAs have also been shown to interact with miRNAs, thereby reversing the effects of miRNAs on mRNA expression. This miRNA sponge role has lncRNAs poised to serve as a tool in controlling miRNA function, potentially in a therapeutic setting [56]. Further, genetic variation in lncRNAs may impact lncRNA expression levels, splicing, and/or the stability of any lncRNA–mRNA interactions [57,58]. While work in model organisms has progressed steadily, the repertoire of lncRNAs in non-model organisms have only recently begun to be explored.

lncRNAs can be cataloged from standard RNA-Seq high-throughput sequencing approaches (detailed methodological and bioinformatic approaches for insect lncRNA discovery reviewed in [59]). Given that lncRNAs tend to be rare compared to mRNA [60], the depth of sequencing necessary to reliably detect lncRNAs should be considered when planning an experiment. Two published studies aimed at cataloging lncRNAs in *Anophelines* used 223 and 500 million sequence reads [61,62]. As many lncRNAs are expressed antisense to protein-coding genes which they often regulate, it is also recommended to employ stranded RNA-seq approaches. To catalog lncRNAs in *Anophelines*, the following data analysis pipeline was employed, TopHat [63] was used for read mapping, Cufflinks [64] for annotation, and CuffCompare for comparison with existing genome annotations. Only transcripts with class codes, “i”, “u”, and “x” denoting intronic, intergenic, and antisense, respectively, were selected as possible lncRNAs. Following mapping and annotation, coding potential was analyzed using one of the available tools, including the Coding Potential Assessment Tool (CPAT) [65], the Coding Potential Calculator (CPC) [66], or PhyloCSF, with CSF standing for Codon Substitution Frequencies [67]. In an *Aedes albopictus* study, novel lncRNA loci were identified using FEELnc, a platform that predicts lncRNA using a random forest model trained on multi k-mer frequencies and relaxed open reading frames [68]. Differentially-regulated lncRNAs identified from RNA-seq data can be validated using standard qRT-PCR approaches.

Although some insect lncRNAs have been identified with functional roles over the last decade, the majority of the progress in determining the function of lncRNA has been made in vertebrates [69]. In recent years, studies examining lncRNAs in insects have increased, with much of this work focused on insect development, insecticide resistance, and antiviral defense in insect pests [70]. Through a computational pipeline, thousands of lncRNAs have been identified from RNA-seq data of the diamondback moth [71]. Other recent work in *Drosophila* has highlighted the important role of lncRNAs in development and immunity [72,73].

lncRNAs are known to play a role in sex determination in various organisms, including mammals, fish, crustaceans, and insects [74,75,76,77]. In *Drosophila*, lncRNAs have been implicated in the activation of expression of the sex determination gene Sex-lethal (Sxl) necessary to determine female sex [78]. In *Aedes aegypti*, sex determination is regulated by the male determining locus, M, located in a Y chromosome-like region on chromosome 1. Through recent sequencing efforts, the *A. aegypti* genome assembly and the annotation of this highly-repetitive M locus have drastically improved [79,80]. The improved genome annotation in the M/m sex determination locus highlights a number of putative lncRNA genes. Work to functionally characterize the role of these predicted lncRNAs and their role in sex determination is ongoing. Given efforts to use release of sterile male mosquitoes for vector control, understanding the molecular mechanisms underlying sex determination could be advantageous for efficient enrichment of male mosquitoes.

There are a small number of studies that have both cataloged lncRNAs in mosquitoes and begun to explore their function, particularly as it relates to viral transmission within the *Aedes* genus. Two studies have implicated lncRNAs in host-arboviral interaction. RNAi-mediated knockdown of one lncRNA candidate in *Ae. aegypti* resulted in higher Dengue virus replication [81], and differentially-expressed lncRNAs have been associated with Zika virus infection in *Ae. aegypti* [82]. A recent cataloging of lncRNAs in *Ae. aegypti* reported that they shared many of their characteristics with lncRNAs from other species, including low levels of expression, low GC content, short length, and less conservation than protein-coding mRNAs [83]. This catalog also highlights that *Ae. aegypti* lncRNAs contain a greater fraction of repeat elements as compared to protein-coding mRNAs, and that lncRNAs display highly temporal expression patterns [83]. Recently the same research team did a similar study in the Southern house mosquito (*Culex quinquefasciatus*) and showed that lncRNAs may play a role in blood meal acquisition in adult females [84]. Work in the *Anopheles* genus has similar findings to work in *Ae. aegypti*, including lower sequence conservation in lncRNAs as compared to protein-coding genes, however there is notable conservation in lncRNA secondary structure within the *Gambiae* complex containing the major malaria vectors in Sub-Saharan Africa, and more divergent secondary structure in the rest of the *Anopheles* genus [61]. A recent study in *Ae. albopictus* identified 2632 novel lncRNAs with a small fraction of these showing male- and female-specific expression patterns [85]. Work on lncRNAs in mosquitoes remains relatively novel and as a result, nothing is known about the functional consequence of genetic variation in lncRNAs.

## 4. Enhancers

Enhancers are short cis-acting regulatory elements that increase transcriptional levels of target genes by hundreds of fold over the basal level of the core promoter elements [86]. Enhancers control transcriptional activity of a gene, or suite of genes, and are responsible for almost all regulated gene expression in the transcriptome [87,88]. Enhancers can be located near their target gene(s) or megabases distant from the target genes they regulate [89]. Nevertheless, the identities of enhancers and the interacting protein factors that lead to their regulatory function are little known, even in well-studied model genomes [87,88]. An important reason for this is that enhancers cannot yet reliably be predicted by sequence-based algorithms, and until recently, available screening methods were manual and thus limited in scale. Sequence polymorphism of enhancer sequences can cause phenotypic differences, including predisposition to disease, as observed in diverse organisms [2,90,91,92,93]. At least 70–90% of significantly-associated human GWAS SNPs are estimated to lie within functional enhancers [2,4,94]. At the population level, positively-selected variation at enhancers and other non-coding regulatory elements between species or subgroups likely play an important role in differentiation and evolution [95,96], for example, some of the most diverged sequence of the human genome, as compared to great apes, have been classified as functional enhancers [97]. Very little is known about enhancers in mosquito disease vectors, and nothing is known about non-coding variation and vector phenotypes. A recent review provides a comprehensive summary on studying enhancers in non-model insects [98]. For an in-depth review on chromatin structure and function in mosquitoes, including 3D explorations of the genome using the Hi-C high-throughput sequencing approaches to identify topologically associated domains (TADs), see this recent review [99]. Here, the focus is on direct and indirect experimental approaches to catalog mosquito transcriptional enhancers.

### Screening for Enhancers

Despite their known role in gene expression regulation [87,88], until recently there has not been a method for high-throughput, direct, and quantitative screening of DNA sequences for enhancer activity. Indirect screening methods such as ChIP-seq and DNase-seq can infer the presence of enhancers by detecting the open chromatin state correlated with binding of trans-acting factors and histone modification, but do not directly measure enhancer activity [100].

In contrast, functional assays detect enhancers by measuring enhancer activity from a target gene with a measurable readout. The gold standard assay is manual cloning of a candidate enhancer fragment into an expression vector, where the putative enhancer activates a minimal core promoter, driving expression of a luciferase reporter, whose light readout is the measure of enhancer-dependent expression [101]. Enhancers carry the information necessary for their autonomous function, which is preserved even when placed into a heterologous surrounding sequence context such as a reporter plasmid. Self-transcribing active regulatory region sequencing (STARR-seq) assay is a massively parallel reporter assay that detects enhancers directly by their functional properties, querying millions of DNA fragments simultaneously [95,102]. STARR-seq is, in essence, a simultaneous genome-wide luciferase assay, with the exception that it measures enhancer-dependent transcript levels as sequence reads from RNA-seq data, rather than light output due to translated protein.

## 5. Direct Methods for Enhancer Discovery

When the goal of an experiment is to discover enhancers or other cis-regulatory elements (CREs), direct methods of regulatory element discovery are often very useful. Such methods find their origins in luciferase assays, where a single DNA sequence is cloned into a vector containing a luciferase reporter construct. This approach is useful for testing one gene at a time and is still considered the gold standard for determining the enhancer activity of a gene. Work in *Anopheles stephensi* has used a transposon-mediated enhancer detection approach using the Gal4-UAS system, but this approach is labor-intensive and does not explore enhancers on a genome-wide scale [103]. There is growing need in the field to identify regulatory elements and their interactions across the genome. It is nearly impossible to screen enhancer activity on a whole genome scale using single gene luciferase assays, and so efforts to scale up the throughput of luciferase assays brought massively parallel reporter assays (MPRAs) [104,105], which allow for the simultaneous assessment of activity for thousands of enhancers. While an important development in the field, MPRAs have three major drawbacks. First, the MPRA approach uses oligonucleotide arrays to synthesize tested sequences with the maximum length of synthesis limited to 200 bp, rendering the study of enhancers larger than 200 bp infeasible. Second, the insertion of reporter genes into the genome on a large scale often causes substantial positional effects, inhibiting the effectiveness of such assays. Finally, enhancer activity cannot be analyzed quantitatively, as MPRAs provide only binary information results (active/inactive) [105].

### STARR-Seq

Self-transcribing active regulatory region sequencing (STARR-seq) is a method of directly discovering and quantitively assessing enhancer activity on a genome-wide scale. STARR-seq identifies active, chromatin-masked, and dormant enhancers by assaying enhancer activity of genomic fragments episomally. Briefly, genomic DNA is fragmented, and linkers are added to fragment ends. This library of fragments is then cloned into a vector downstream of a core promoter, the vector library is transfected into cells, and after 24 h, RNA is harvested, and a cDNA library generated. Genomic DNA is simultaneously harvested to control for differential transfection efficiencies. Cloned fragments with enhancer activity will drive expression of themselves and resulting sequence output will both identify enhancers and quantify their activity. This method allows for the simultaneous screening of the entire genome for enhancer activity [95,102,106]. There are a number of available methods for analysis of STARR-seq data and identification of enhancer peaks [107,108]. Drawbacks of STARR-seq are twofold; the first being that many enhancers are “context dependent”, meaning that their position in the genome is important, and the STARR-seq approach removes DNA fragments from their genomic context. Enhancers may interact with other nearby regulatory elements, or distal regulatory elements that are brought to interact with an enhancer through changes in the chromatin structure. The second being that this method discovers all enhancers within the tested DNA, making it difficult to determine which enhancers are relevant to a condition [105]. Despite these limitations, this method, capitalizing on next-generation sequencing approaches to comprehensively query enhancer activity on a genome-wide scale, generates a comprehensive catalog of an organism’s enhancers.

STARR-seq has been used in *Drosophila* to comprehensively characterize and compare transcriptional enhancers across five closely-related species [95]. This seminal work concludes that there is a good degree of evolutionary conservation in enhancer activity, as well as frequent gains in enhancer function since divergence from the common ancestor. Work in *An. coluzzii* has examined the impact of naturally-segregating genetic variation in a small number of enhancers with potential roles in mosquito development, immunity, and insecticide resistance [109].

## 6. Indirect Methods for Enhancer Discovery

Indirect approaches to discovering cis-regulatory elements operate through the detection of open chromatin. These indirect methods are predicated on the knowledge that active regulatory elements exist within open chromatin. A variety of methods now exist that either tag or remove open DNA (or both tag and remove), allowing this portion of the genomic DNA to be selectively sequenced. As chromatin can be open both constitutively and conditionally, an indirect approach to regulatory element discovery is valuable in the detection of genomic structural changes that may impact gene expression. This section explores a number of indirect methods for the detection of enhancers/cis-regulatory elements.

### 6.1. ATAC-Seq (Single Cell Capable)

Assay for transposase-accessible chromatin using sequencing (ATAC-seq) is one method of detecting open chromatin and enhancers. This method employs enzymatic manipulation of DNA, specifically using a hyperactive Tn5 transposase to cut and “tag” open DNA with adaptor sequences [110]. The method has gained increasing popularity due to its need for small amounts of input DNA and shorter experimental run time (less than three days) [111]. As mentioned previously, the open DNA sequence is bound to a hyperactive derivative of Tn5 which is flanked by 19 bp sequences called mosaic ends (MEs). These MEs are specific to the sequence around the insertion-site DNA. The open DNA is subsequently cut by Tn5 transposase derivatives, and the MEs remain attached, tagging the cut DNA with a specific sequence [112]. This “tagmented” DNA is subsequently purified, amplified, and sequenced. ATAC-seq is a method that can be done at the scale of the single cell, which affords very fine-scale characterization (see review of the single cell approach here [113]). While ATAC-seq has become a more commonly-used approach in the last five years, the method has its own set of drawbacks, including the amplification of non-nuclear, particularly mitochondrial DNA [110,114]. Methods for analysis of ATAC-seq data are evolving, and a recent publication provides an up-to-date review of current methods, including quality control steps, peak identification, and identification of differential peaks [115].

Use of ATAC-seq in mosquitoes is limited to two studies on *Ae. aegypti* and *An. gambiae* [80,116]. In *Ae. aegypti*, the method was adapted from the original protocol published in 2013 [111,117] for use on *Ae. aegypti* brains to map CRE at predicted transcription start sites in the updated genome, AaegL5 [80]. In *An. gambiae*, genome-wide profiling of chromatin accessibility was done using the salivary glands and midguts of *Plasmodium*-infected females. ATAC-seq was used in combination with RNA-seq and ChIP-seq data to demonstrate that chromatin accessibility was greatest in promoter regions and introns, and that these open regions also correlated with tissue-specific gene expression. The study identified potentially important regulatory regions within the *An. gambiae* genome [116].

### 6.2. ChIP-Seq

Chromatin immunoprecipitation (ChIP-seq) is another method of detecting open chromatin, and thereby indirectly cataloging CREs. ChIP-seq starts with resident proteins being crosslinked to the DNA. DNA is then sheared using sonication, incubated with antibodies, and immunoprecipitated. Immunoprecipitated DNA is then amplified and sequenced [118]. ChIP-seq is the oldest method for cis-regulatory element/enhancer discovery, and has reliably produced high-resolution results [118]. However, ChIP-seq can be difficult to use for some laboratories or with some organisms, due to its high time cost, its need for large amounts of DNA, and the need for highly-specific antibodies that are not always readily available [114,119].

In *An. gambiae*, ChIP-seq combined with RNA-seq have been used to study the chromatin modifications accompanying *Plasmodium* infection [120]. Most of this work used histone modification markers known to associate with promoters. A comprehensive look at enhancers would require the use of chromatin marks, such as H_3_K_4_me1, known to be enhancer-associated [120]. Earlier work in *An. gambiae* cemented a correlation between the histone modification marks, H_3_K_27_ac and H_3_K_27_me3, and increased/decreased gene expression, respectively [121]. Work done by Lukyanchikova et al. examined the 3D architecture of five *Anopheline* mosquito species [122]. Much of the analysis was performed using Hi-C to examine new looping interactions in the 3D genome. Chromatin loops had been previously associated with the polycomb group of proteins that largely function in maintaining cell positional identity. To examine this association, ChIP-seq was performed on *Anopheles atroparvus,* revealing that some of the looping structures were anchored in H_3_K_27_me3-enriched silencing regions. ChIP-seq has also been used in *Cu. pipiens* to determine 72 new targets of the forkhead transcription factor (FOXO) [123]. Two important signaling pathways appear to hinge on the presence of FOXO in order to transition adult *Cu. pipiens* mosquitoes into their overwinter diapause. Discovery of these new target genes represent an expansion in the previous knowledge of FOXO interactions.

### 6.3. DNase-Seq (Single Cell Capable)

DNase-seq is the second oldest method of indirect regulatory element discovery and is another reliable, well-documented method [124]. DNase-seq finds its roots in DNase-footprinting, a technique that similarly uses DNase I to digest DNA but culminates in a gel electrophoresis step, relying on DNA-fragment sizes to report the “footprint” of binding proteins [125]. DNase-seq advances the merits of its predecessor by providing an even more detailed initial look at chromatin structure where there may have been no previous understanding. These advances lie in the coupling of DNase-footprinting with high-throughput sequencing approaches [126]. The method uses slightly less DNA than ChIP-seq, but there is a risk of enzymatic cleavage bias that may skew the results [114]. DNase-seq begins with a Dnase I chromatin digestion where open DNA is selectively excised. This cleavage reaction is stopped when it is loaded onto a low-melt agarose gel and subjected to electrophoresis. The desired bands are removed from the gel, and the open DNA within them is amplified and sequenced by high-throughput sequencing technologies [114,126,127]. There are no published uses of DNase-seq in mosquito disease vectors, and only one published use of DNAse-footprinting [128].

### 6.4. FAIRE-Seq

Formaldehyde-assisted isolation of regulatory elements sequencing (FAIRE-seq) is a method of indirect regulatory element discovery commonly used due to its straightforward application, low cleavage bias, and its ability to be applied to many different cell types [126]. There are three basic steps to FAIRE-seq: first, the DNA, similar to in ChIP-seq, is crosslinked and sheared; second, the non-crosslinked DNA is phenol-chloroform-extracted and third, this DNA is then amplified and sequenced [129,130]. Though the procedure is straightforward, FAIRE-seq generates a low signal-to-noise ratio that can make data processing difficult. Additionally, the variable length of time required for the formaldehyde fixation step can make it hard to plan experiment time effectively [126,129,131].

FAIRE-seq was used to generate a genome-wide map of regulatory elements in *Ae. aegypti*. Very interestingly, of the large number of single nucleotide polymorphisms identified in mosquito strains susceptible and resistant to dengue virus, more than a quarter of these SNPs overlap with regulatory peaks, suggesting that variation in regulatory sequences can contribution to variability in the susceptibility to dengue infection [131].

In *An. gambiae*, FAIRE-seq was used in a study of cis-regulatory elements involved in innate immune function. Sequences for new CREs were discovered and may prove useful in predicting protein–protein interactions in the *An. gambiae* immune responses [132].

### 6.5. MNase-Seq (Single Cell Capable)

MNase-seq, while also an indirect method of enhancer identification, is different in that it does not involve fragmenting open chromatin, but rather is designed to cleave and degrade internucleosomal DNA [110]. Micrococcal nuclease (MNase) is an endo-exonuclease derived from *Staphylococcus aureus*, and its first use, to determine chromatin structure, dates back to 1975 [133]. The first instance of MNase paired with high-throughput sequencing, however, was in 2009 [134]. The technique begins with the digestion of genomic DNA with MNase to extract mononucleosomes. Following this, the DNA from the DNA-protein complexes are extracted and used to prepare a sequencing library. High-throughput sequencing then provides the genomic location of regulatory DNA-binding proteins in the genome [126]. One drawback of MNase-seq is the potentially variable digestion of MNase. Activity of the enzyme can be highly dependent on MNase concentration, making results potentially highly variable, even across experimental replicates [110,126,135]. There are no published uses of MNase-seq in mosquito disease vectors. MNase-seq has been used in the malaria parasite, *Plasmodium falciparum* to study the role of nucleosome positioning in the regulation of gene transcription [136]. In *Drosophila*, MNase-seq has been used to track changes in the nucleosome occupancy in response to immune stimulation [137].

### 6.6. NOMe-Seq

Nucleosome occupancy and methylome sequencing (NOMe-seq) is a more recent technique that is notable for its ability to both detect nucleosomes occupancy and methylation patterns in DNA [138]. NOMe-seq is performed by fixing cells and shearing the DNA to >1 kb fragments. The enzyme M.CviPI is then used to methylate unprotected GC dinucleotides in accessible DNA. Next, a bisulfite conversion is performed to convert all unmethylated cytosine into uracil. The prepared DNA is then purified, amplified, and sequenced [138]. NOMe-seq finds its one drawback in the need for specific DNA fragment sizes to prevent bias towards CpG islands [139]. There are no published uses of DNase-seq in mosquito disease vectors or in other insects. In the nearly 10 years since it was first introduced, there are only 19 published papers using this method (see Table 1).

## 7. Enhancer RNAs

Distinct from the enhancers detected by either indirect and direct methods are enhancer RNAs (eRNAs), which are non-coding RNA molecules transcribed from enhancer regions of the genome and comprises two main classes, 1D eRNAs and 2D eRNAs. These two classes of eRNAs differ in size, polyadenylation, and direction of transcription. 1D eRNAs are 3–4 kb in length, polyadenylated, and unidirectional, while 2D eRNAs are typically less than 2 kb, nonpolyadenylated, and bidirectional [142]. The functional role of eRNAs is not well characterized [143], but there does appear to be an association between eRNA expression and enhancer activity [144]. Previous data would suggest that eRNAs are able to self-transcribe and act as transcription factor complexes both in cis and trans, and may be necessary to help chromatin maintain its open state [145]. The process for discovering eRNAs generally includes two steps, the use of an indirect CRE discovery method, such as ChIP-seq, coupled with RNA-seq. Nothing is known about eRNAs in mosquitoes, but they likely play an important role in the regulation of gene expression in *Drosophila* [146]. 

## 8. Concluding Remarks

With continuously improving technological approaches, efforts to functionally characterize the non-coding genome in mosquito disease vectors are advancing. With regulatory elements such as miRNAs, lncRNAs, and enhancers identified and cataloged, efforts will shift to characterizing the functional consequence of genetic variation in these elements. A combination of direct and indirect experimental approaches will generate the most comprehensive picture of non-coding regulatory elements, their dynamic interactions with coding elements, and their impact on organism phenotype. Efforts such as the large-scale Ag1000 genomes sequencing project [147] will also aide in cataloging naturally-segregating variation in these non-coding regions.

## Figures and Tables

**Table 1:** Direct and indirect methods for enhancers/cis-regulatory element discovery.

Method	Date of First Publication ^a^	Protocol	Time Needed ^c^	Number of Mosquitoes Needed ^d^	Previous Use in Mosquito Disease Vectors	Protocol Bias
**ATAC-seq** Insert known sequence tags into open DNA	**2013** [117] (543 ref)	Tagmentation DNA purification DNA labeling Sequencing	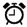	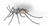	*Aedes aegypti:* [80] *Anopheles gambiae:* [116]	Generates non-specific amplification of extra-nuclear DNA (mitochondrial)[114]
**CHIP-seq** Immunoprecipitate open DNA	**2007** [140] (4382 ref)	Crosslink proteins to DNA Shear DNA Immunoprecipitation of open DNA Sequencing	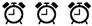	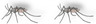	*Anopheles atroparvus:* [122] *Culex pipiens:* [123] *Anopheles gambiae:* [120,121]	Antibody availability and specificity [114]
**DNase-seq** Enzymatically remove open DNA	**2008** [124] (194 ref)	DNaseI DNA digestion Gel electrophoresis Sequencing	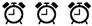	ND	None	Dnase I cleavage bias [127]
**FAIRE-seq** Crosslinking and extracting open DNA	**2009** [130] (60 ref)	DNA crosslinked and sheared Phenol-Chloroform extraction Sequencing	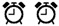	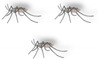	*Aedes aegypti:* [131] *Anopheles gambiae:* [132]	Low signal to noise ratio, variation in formaldehyde fixation step [126]
**MNase-seq** Enzymatically remove nucleosomal DNA	**2009** [134] (90 ref)	Mnase DNA digestion Nucleosomal DNA purified Sequencing	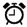	ND	None	Variable Mnase digestion [126]
**NOMe-seq** Methylate accessible DNA	**2011** [141] (19 ref)	Cells fixed and sheared M.CvPi ^b^ methylation of GC dinucleotides Bisulfite Conversion Sequencing	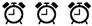	ND	None	Requires specific library fragment size to minimize bias towards CpG islands [139]
**STARR-seq** Quantitatively assesses enhancer activity of genomic fragments on a genome-wide scale	**2013** [102] (27 ref)	Genomic DNA fragmented Addition of linkers Cloned into vector Cell transfection mRNA isolation and cDNA generation Sequencing	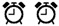	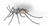	None	Does not capture conditional states, catalogs all enhancers [105]

^a^ Date of first and total references were determined in Dec 2020 by searching PubMed using the protocol name in quotations, followed by the field term that searches the title and abstract for the protocol name: [TIAB] (i.e., “ATAC-seq” [TIAB]); ^b^ M.CviPI is an enzyme that methylates GpC dinucleotides; ^c^ One clock signifies <3 days of wet lab work; two clocks, 3 days of wet work, and three clocks, >3 days of wet work; ^d^ One mosquito indicates <100 individual mosquitoes used in a single experimental sample; two mosquitoes, 100 individuals, and three mosquitoes, >100 individuals; ND = not determined.

## Data Availability

Not applicable.

## References

[1] Ohno S., Smith H.H. (1972). So much “junk” DNA in our genome. Evolution of Genetic Systems.

[2] Farh K.K., Marson A., Zhu J., Kleinewietfeld M., Housley W.J., Beik S., Shoresh N., Whitton H., Ryan R.J., Shishkin A.A. (2015). Genetic and epigenetic fine mapping of causal autoimmune disease variants. Nature.

[3] Altshuler D.M., Gibbs R.A., Peltonen L., Altshuler D.M., Gibbs R.A., Peltonen L., Dermitzakis E., Schaffner S.F., Yu F., The International HapMap 3 Consortium (2010). Integrating common and rare genetic variation in diverse human populations. Nature.

[4] Neph S., Vierstra J., Stergachis A.B., Reynolds A.P., Haugen E., Vernot B., Thurman R.E., John S., Sandstrom R., Johnson A.K. (2012). An expansive human regulatory lexicon encoded in transcription factor footprints. Nature.

[5] Ayala D., Zhang S., Chateau M., Fouet C., Morlais I., Costantini C., Hahn M.W., Besansky N. (2018). Association mapping desiccation resistance within chromosomal inversions in the African malaria vector Anopheles gambiae. Mol. Ecol..

[6] Kolliopoulou A., Santos D., Taning C.N.T., Wynant N., Vanden Broeck J., Smagghe G., Swevers L. (2019). PIWI pathway against viruses in insects. Wiley Interdiscip Rev. RNA.

[7] Zhu K.Y., Palli S.R. (2020). Mechanisms, Applications, and Challenges of Insect RNA Interference. Annu. Rev. Entomol..

[8] Ozata D.M., Gainetdinov I., Zoch A., O’Carroll D., Zamore P.D. (2019). PIWI-interacting RNAs: Small RNAs with big functions. Nat. Rev. Genet..

[9] Bartel D.P. (2004). MicroRNAs: Genomics, biogenesis, mechanism, and function. Cell.

[10] Carthew R.W., Sontheimer E.J. (2009). Origins and Mechanisms of miRNAs and siRNAs. Cell.

[11] Biggar K.K., Storey K.B. (2018). Functional impact of microRNA regulation in models of extreme stress adaptation. J. Mol. Cell Biol..

[12] Feng X., Zhou S., Wang J., Hu W. (2018). microRNA profiles and functions in mosquitoes. PLoS Negl. Trop. Dis..

[13] Schuster S., Miesen P., van Rij R.P. (2019). Antiviral RNAi in Insects and Mammals: Parallels and Differences. Viruses.

[14] Hutvágner G., Zamore P.D. (2002). A microRNA in a multiple-turnover RNAi enzyme complex. Science.

[15] Martinez J., Tuschl T. (2004). RISC is a 5′ phosphomonoester-producing RNA endonuclease. Genes Dev..

[16] Bushati N., Cohen S.M. (2007). microRNA Functions. Annu. Rev. Cell Dev. Biol..

[17] Fabian M.R., Sonenberg N. (2012). The mechanics of miRNA-mediated gene silencing: A look under the hood of miRISC. Nat. Struct. Mol. Biol..

[18] Bartel D.P. (2009). MicroRNAs: Target recognition and regulatory functions. Cell.

[19] Bhartiya D., Scaria V. (2016). Genomic variations in non-coding RNAs: Structure, function and regulation. Genomics.

[20] Bensen J.T., Graff M., Young K.L., Sethupathy P., Parker J., Pecot C.V., Currin K., Haddad S.A., Ruiz-Narvaez E.A., Haiman C.A. (2018). A survey of microRNA single nucleotide polymorphisms identifies novel breast cancer susceptibility loci in a case-control, population-based study of African-American women. Breast Cancer Res..

[21] Carissimo G., Pain A., Belda E., Vernick K.D. (2018). Highly focused transcriptional response of Anopheles coluzzii to O’nyong nyong arbovirus during the primary midgut infection. BMC Genom..

[22] Lampe L., Levashina E.A. (2017). The role of microRNAs in Anopheles biology-an emerging research field. Parasite Immunol..

[23] Dennison N.J., BenMarzouk-Hidalgo O.J., Dimopoulos G. (2015). MicroRNA-regulation of Anopheles gambiae immunity to Plasmodium falciparum infection and midgut microbiota. Dev. Comp. Immunol..

[24] Ling L., Kokoza V.A., Zhang C., Aksoy E., Raikhel A.S. (2017). MicroRNA-277 targets insulin-like peptides 7 and 8 to control lipid metabolism and reproduction in Aedes aegypti mosquitoes. Proc. Natl. Acad. Sci. USA.

[25] Git A., Dvinge H., Salmon-Divon M., Osborne M., Kutter C., Hadfield J., Bertone P., Caldas C. (2010). Systematic comparison of microarray profiling, real-time PCR, and next-generation sequencing technologies for measuring differential microRNA expression. RNA.

[26] Martin J.A., Wang Z. (2011). Next-generation transcriptome assembly. Nat. Rev. Genet..

[27] Kang W., Friedländer M.R. (2015). Computational Prediction of miRNA Genes from Small RNA Sequencing Data. Front. Bioeng. Biotechnol..

[28] Haac M.E., Anderson M.A., Eggleston H., Myles K.M., Adelman Z.N. (2015). The hub protein loquacious connects the microRNA and short interfering RNA pathways in mosquitoes. Nucleic Acids Res..

[29] Liu W., Hao Z., Huang L., Chen L., Wei Q., Cai L., Liang S. (2017). Comparative expression profile of microRNAs in Anopheles anthropophagus midgut after blood-feeding and Plasmodium infection. Parasites Vectors.

[30] Raabe C.A., Tang T.H., Brosius J., Rozhdestvensky T.S. (2014). Biases in small RNA deep sequencing data. Nucleic Acids Res..

[31] Witwer K.W., Halushka M.K. (2016). Toward the promise of microRNAs-Enhancing reproducibility and rigor in microRNA research. RNA Biol..

[32] Chen L., Heikkinen L., Wang C., Yang Y., Sun H., Wong G. (2019). Trends in the development of miRNA bioinformatics tools. Brief. Bioinform..

[33] Samuel G.H., Adelman Z.N., Myles K.M. (2018). Antiviral Immunity and Virus-Mediated Antagonism in Disease Vector Mosquitoes. Trends Microbiol..

[34] Li S., Mead E.A., Liang S., Tu Z. (2009). Direct sequencing and expression analysis of a large number of miRNAs in Aedes aegypti and a multi-species survey of novel mosquito miRNAs. BMC Genom..

[35] Osei-Amo S., Hussain M., O’Neill S.L., Asgari S. (2012). Wolbachia-induced aae-miR-12 miRNA negatively regulates the expression of MCT1 and MCM6 genes in Wolbachia-infected mosquito cell line. PLoS ONE.

[36] Jain S., Rana V., Shrinet J., Sharma A., Tridibes A., Sunil S., Bhatnagar R.K. (2014). Blood feeding and Plasmodium infection alters the miRNome of Anopheles stephensi. PLoS ONE.

[37] Avila-Bonilla R.G., Yocupicio-Monroy M., Marchat L.A., Pérez-Ishiwara D.G., Cerecedo-Mercado D.A., Del Ángel R.M., Salas-Benito J.S. (2020). miR-927 has pro-viral effects during acute and persistent infection with dengue virus type 2 in C6/36 mosquito cells. J. Gen. Virol..

[38] Su J., Wang G., Li C., Xing D., Yan T., Zhu X., Liu Q., Wu Q., Guo X., Zhao T. (2019). Screening for differentially expressed miRNAs in Aedes albopictus (Diptera: Culicidae) exposed to DENV-2 and their effect on replication of DENV-2 in C6/36 cells. Parasit Vectors.

[39] Dubey S.K., Shrinet J., Sunil S. (2019). Aedes aegypti microRNA, miR-2944b-5p interacts with 3’UTR of chikungunya virus and cellular target vps-13 to regulate viral replication. PLoS Negl. Trop. Dis..

[40] Trobaugh D.W., Sun C., Bhalla N., Gardner C.L., Dunn M.D., Klimstra W.B. (2019). Cooperativity between the 3’ untranslated region microRNA binding sites is critical for the virulence of eastern equine encephalitis virus. PLoS Pathog..

[41] Yen P.S., Chen C.H., Sreenu V., Kohl A., Failloux A.B. (2019). Assessing the Potential Interactions between Cellular miRNA and Arboviral Genomic RNA in the Yellow Fever Mosquito, Aedes aegypti. Viruses.

[42] Sinclair J.B., Asgari S. (2020). Ross River Virus Provokes Differentially Expressed MicroRNA and RNA Interference Responses in Aedes aegypti Mosquitoes. Viruses.

[43] Lampe L., Jentzsch M., Kierszniowska S., Levashina E.A. (2019). Metabolic balancing by miR-276 shapes the mosquito reproductive cycle and Plasmodium falciparum development. Nat. Commun..

[44] Lampe L., Levashina E.A. (2018). MicroRNA Tissue Atlas of the Malaria Mosquito Anopheles gambiae. G3 (Bethesda).

[45] Meuti M.E., Bautista-Jimenez R., Reynolds J.A. (2018). Evidence that microRNAs are part of the molecular toolkit regulating adult reproductive diapause in the mosquito, Culex pipiens. PLoS ONE.

[46] Moore M.J., Scheel T.K., Luna J.M., Park C.Y., Fak J.J., Nishiuchi E., Rice C.M., Darnell R.B. (2015). miRNA-target chimeras reveal miRNA 3′-end pairing as a major determinant of Argonaute target specificity. Nat. Commun..

[47] Dong S., Fu X., Dong Y., Simões M.L., Zhu J., Dimopoulos G. (2020). Broad spectrum immunomodulatory effects of Anopheles gambiae microRNAs and their use for transgenic suppression of Plasmodium. PLoS Pathog..

[48] Fu X., Liu P., Dimopoulos G., Zhu J. (2020). Dynamic miRNA-mRNA interactions coordinate gene expression in adult Anopheles gambiae. PLOS Genet..

[49] Zhang Y., Zhao B., Roy S., Saha T.T., Kokoza V.A., Li M., Raikhel A.S. (2016). microRNA-309 targets the Homeobox gene SIX4 and controls ovarian development in the mosquito Aedes aegypti. Proc. Natl. Acad. Sci. USA.

[50] Derrien T., Johnson R., Bussotti G., Tanzer A., Djebali S., Tilgner H., Guernec G., Martin D., Merkel A., Knowles D.G. (2012). The GENCODE v7 catalog of human long noncoding RNAs: Analysis of their gene structure, evolution, and expression. Genome Res..

[51] Liu S.J., Nowakowski T.J., Pollen A.A., Lui J.H., Horlbeck M.A., Attenello F.J., He D., Weissman J.S., Kriegstein A.R., Diaz A.A. (2016). Single-cell analysis of long non-coding RNAs in the developing human neocortex. Genome Biol..

[52] Mercer T.R., Dinger M.E., Sunkin S.M., Mehler M.F., Mattick J.S. (2008). Specific expression of long noncoding RNAs in the mouse brain. Proc. Natl. Acad. Sci. USA.

[53] Bonasio R., Shiekhattar R. (2014). Regulation of transcription by long noncoding RNAs. Annu. Rev. Genet..

[54] Gardini A., Shiekhattar R. (2015). The many faces of long noncoding RNAs. FEBS J..

[55] Ulitsky I., Bartel D.P. (2013). lincRNAs: Genomics, evolution, and mechanisms. Cell.

[56] Militello G., Weirick T., John D., Doring C., Dimmeler S., Uchida S. (2017). Screening and validation of lncRNAs and circRNAs as miRNA sponges. Brief Bioinform..

[57] Schmitt A.M., Chang H.Y. (2016). Long Noncoding RNAs in Cancer Pathways. Cancer Cell.

[58] Shastry B.S. (2009). SNPs: Impact on gene function and phenotype. Methods Mol. Biol..

[59] Legeai F., Derrien T. (2015). Identification of long non-coding RNAs in insects genomes. Curr. Opin. Insect Sci..

[60] Maciel L.F., Morales-Vicente D.A., Silveira G.O., Ribeiro R.O., Olberg G.G.O., Pires D.S., Amaral M.S., Verjovski-Almeida S. (2019). Weighted Gene Co-Expression Analyses Point to Long Non-Coding RNA Hub Genes at Different Schistosoma mansoni Life-Cycle Stages. Front. Genet..

[61] Jenkins A.M., Waterhouse R.M., Muskavitch M.A. (2015). Long non-coding RNA discovery across the genus anopheles reveals conserved secondary structures within and beyond the Gambiae complex. BMC Genom..

[62] Padron A., Molina-Cruz A., Quinones M., Ribeiro J.M., Ramphul U., Rodrigues J., Shen K., Haile A., Ramirez J.L., Barillas-Mury C. (2014). In depth annotation of the Anopheles gambiae mosquito midgut transcriptome. BMC Genom..

[63] Trapnell C., Pachter L., Salzberg S.L. (2009). TopHat: Discovering splice junctions with RNA-Seq. Bioinformatics.

[64] Trapnell C., Williams B.A., Pertea G., Mortazavi A., Kwan G., van Baren M.J., Salzberg S.L., Wold B.J., Pachter L. (2010). Transcript assembly and quantification by RNA-Seq reveals unannotated transcripts and isoform switching during cell differentiation. Nat. Biotechnol..

[65] Wang L., Park H.J., Dasari S., Wang S., Kocher J.P., Li W. (2013). CPAT: Coding-Potential Assessment Tool using an alignment-free logistic regression model. Nucleic Acids Res..

[66] Kong L., Zhang Y., Ye Z.Q., Liu X.Q., Zhao S.Q., Wei L., Gao G. (2007). CPC: Assess the protein-coding potential of transcripts using sequence features and support vector machine. Nucleic Acids Res..

[67] Lin M.F., Jungreis I., Kellis M. (2011). PhyloCSF: A comparative genomics method to distinguish protein coding and non-coding regions. Bioinformatics.

[68] Wucher V., Legeai F., Hedan B., Rizk G., Lagoutte L., Leeb T., Jagannathan V., Cadieu E., David A., Lohi H. (2017). FEELnc: A tool for long non-coding RNA annotation and its application to the dog transcriptome. Nucleic Acids Res..

[69] Mei-zhen L., Hua-mei X., Kang H., Fei L. (2019). Progress and prospects of noncoding RNAs in insects. J. Integr. Agric..

[70] Wang Y., Xu T., He W., Shen X., Zhao Q., Bai J., You M. (2018). Genome-wide identification and characterization of putative lncRNAs in the diamondback moth, *Plutella xylostella* (L.). Genomics.

[71] Etebari K., Furlong M.J., Asgari S. (2015). Genome wide discovery of long intergenic non-coding RNAs in Diamondback moth (*Plutella xylostella*) and their expression in insecticide resistant strains. Sci. Rep..

[72] Chen M.J., Chen L.K., Lai Y.S., Lin Y.Y., Wu D.C., Tung Y.A., Liu K.Y., Shih H.T., Chen Y.J., Lin Y.L. (2016). Integrating RNA-seq and ChIP-seq data to characterize long non-coding RNAs in Drosophila melanogaster. BMC Genom..

[73] Young R.S., Marques A.C., Tibbit C., Haerty W., Bassett A.R., Liu J.L., Ponting C.P. (2012). Identification and properties of 1119 candidate lincRNA loci in the Drosophila melanogaster genome. Genome Biol. Evol..

[74] Feng X., Wu J., Zhou S., Wang J., Hu W. (2018). Characterization and potential role of microRNA in the Chinese dominant malaria mosquito Anopheles sinensis (Diptera: Culicidae) throughout four different life stages. Cell Biosci..

[75] Hansen T.B., Jensen T.I., Clausen B.H., Bramsen J.B., Finsen B., Damgaard C.K., Kjems J. (2013). Natural RNA circles function as efficient microRNA sponges. Nature.

[76] Kato Y., Perez C.A.G., Mohamad Ishak N.S., Nong Q.D., Sudo Y., Matsuura T., Wada T., Watanabe H. (2018). A 5′ UTR-Overlapping LncRNA Activates the Male-Determining Gene doublesex1 in the Crustacean Daphnia magna. Curr. Biol..

[77] Wu Y., Cheng T., Liu C., Liu D., Zhang Q., Long R., Zhao P., Xia Q. (2016). Systematic Identification and Characterization of Long Non-Coding RNAs in the Silkworm, Bombyx mori. PLoS ONE.

[78] Mulvey B.B., Olcese U., Cabrera J.R., Horabin J.I. (2014). An interactive network of long non-coding RNAs facilitates the Drosophila sex determination decision. Biochim. Biophys. Acta.

[79] Giraldo-Calderon G.I., Emrich S.J., MacCallum R.M., Maslen G., Dialynas E., Topalis P., Ho N., Gesing S., VectorBase C., Madey G. (2015). VectorBase: An updated bioinformatics resource for invertebrate vectors and other organisms related with human diseases. Nucleic Acids Res..

[80] Matthews B.J., Dudchenko O., Kingan S.B., Koren S., Antoshechkin I., Crawford J.E., Glassford W.J., Herre M., Redmond S.N., Rose N.H. (2018). Improved reference genome of Aedes aegypti informs arbovirus vector control. Nature.

[81] Etebari K., Asad S., Zhang G., Asgari S. (2016). Identification of Aedes aegypti Long Intergenic Non-coding RNAs and Their Association with Wolbachia and Dengue Virus Infection. PLoS Negl. Trop. Dis..

[82] Etebari K., Hegde S., Saldana M.A., Widen S.G., Wood T.G., Asgari S., Hughes G.L. (2017). Global Transcriptome Analysis of Aedes aegypti Mosquitoes in Response to Zika Virus Infection. mSphere.

[83] Azlan A., Obeidat S.M., Yunus M.A., Azzam G. (2019). Systematic identification and characterization of Aedes aegypti long noncoding RNAs (lncRNAs). Sci. Rep..

[84] Azlan A., Halim M.A., Mohamad F., Azzam G. (2020). Identification and characterization of long noncoding RNAs and their association with acquisition of blood meal in Culex quinquefasciatus. Insect Sci..

[85] Xu Y., Dong Y., Xu Y., Lai Z., Jin B., Hao Y., Gao Y., Sun Y., Chen X.G., Gu J. (2019). Differentiation of Long Non-Coding RNA and mRNA Expression Profiles in Male and Female Aedes albopictus. Front. Genet..

[86] Shlyueva D., Stampfel G., Stark A. (2014). Transcriptional enhancers: From properties to genome-wide predictions. Nat. Rev. Genet..

[87] Blackwood E.M., Kadonaga J.T. (1998). Going the distance: A current view of enhancer action. Science.

[88] Pennacchio L.A., Bickmore W., Dean A., Nobrega M.A., Bejerano G. (2013). Enhancers: Five essential questions. Nat. Rev. Genet..

[89] Catarino R.R., Neumayr C., Stark A. (2017). Promoting transcription over long distances. Nat. Genet..

[90] Kharchenko P.V., Alekseyenko A.A., Schwartz Y.B., Minoda A., Riddle N.C., Ernst J., Sabo P.J., Larschan E., Gorchakov A.A., Gu T. (2011). Comprehensive analysis of the chromatin landscape in Drosophila melanogaster. Nature.

[91] Romanoski C.E., Link V.M., Heinz S., Glass C.K. (2015). Exploiting genomics and natural genetic variation to decode macrophage enhancers. Trends Immunol..

[92] Schaub M.A., Boyle A.P., Kundaje A., Batzoglou S., Snyder M. (2012). Linking disease associations with regulatory information in the human genome. Genome Res..

[93] Sicard A., Kappel C., Lee Y.W., Wozniak N.J., Marona C., Stinchcombe J.R., Wright S.I., Lenhard M. (2016). Standing genetic variation in a tissue-specific enhancer underlies selfing-syndrome evolution in Capsella. Proc. Natl. Acad. Sci. USA.

[94] Mumbach M.R., Satpathy A.T., Boyle E.A., Dai C., Gowen B.G., Cho S.W., Nguyen M.L., Rubin A.J., Granja J.M., Kazane K.R. (2017). Enhancer connectome in primary human cells identifies target genes of disease-associated DNA elements. Nat. Genet..

[95] Arnold C.D., Gerlach D., Spies D., Matts J.A., Sytnikova Y.A., Pagani M., Lau N.C., Stark A. (2014). Quantitative genome-wide enhancer activity maps for five Drosophila species show functional enhancer conservation and turnover during cis-regulatory evolution. Nat. Genet..

[96] Franchini L.F., Pollard K.S. (2015). Can a few non-coding mutations make a human brain?. Bioessays.

[97] Vierstra J., Rynes E., Sandstrom R., Zhang M., Canfield T., Hansen R.S., Stehling-Sun S., Sabo P.J., Byron R., Humbert R. (2014). Mouse regulatory DNA landscapes reveal global principles of cis-regulatory evolution. Science.

[98] Tomoyasu Y., Halfon M.S. (2020). How to study enhancers in non-traditional insect models. J. Exp. Biol..

[99] Lezcano O.M., Sánchez-Polo M., Ruiz J.L., Gómez-Díaz E. (2020). Chromatin Structure and Function in Mosquitoes. Front. Genet..

[100] Wold B., Myers R.M. (2008). Sequence census methods for functional genomics. Nat. Methods.

[101] Gould S.J., Subramani S. (1988). Firefly luciferase as a tool in molecular and cell biology. Anal. Biochem..

[102] Arnold C.D., Gerlach D., Stelzer C., Boryn L.M., Rath M., Stark A. (2013). Genome-wide quantitative enhancer activity maps identified by STARR-seq. Science.

[103] O’Brochta D.A., Pilitt K.L., Harrell R.A., Aluvihare C., Alford R.T. (2012). Gal4-based enhancer-trapping in the malaria mosquito Anopheles stephensi. G3 (Bethesda).

[104] Inoue F., Ahituv N. (2015). Decoding enhancers using massively parallel reporter assays. Genomics.

[105] Muerdter F., Boryn L.M., Arnold C.D. (2015). STARR-seq-principles and applications. Genomics.

[106] Neumayr C., Pagani M., Stark A., Arnold C.D. (2019). STARR-seq and UMI-STARR-seq: Assessing Enhancer Activities for Genome-Wide-, High-, and Low-Complexity Candidate Libraries. Curr. Protoc Mol. Biol..

[107] Buerger A. (2020). BasicSTARRseq: Basic Peak Calling on STARR-Seq Data. https://bioconductor.org/packages/release/bioc/html/BasicSTARRseq.html.

[108] Lee D., Shi M., Moran J., Wall M., Zhang J., Liu J., Fitzgerald D., Kyono Y., Ma L., White K.P. (2020). STARRPeaker: Uniform processing and accurate identification of STARR-seq active regions. Genome Biol..

[109] Nardini L., Holm I., Pain A., Bischoff E., Gohl D.M., Zongo S., Guelbeogo W.M., Sagnon N., Vernick K.D., Riehle M.M. (2019). Influence of genetic polymorphism on transcriptional enhancer activity in the malaria vector Anopheles coluzzii. Sci. Rep..

[110] Klemm S.L., Shipony Z., Greenleaf W.J. (2019). Chromatin accessibility and the regulatory epigenome. Nat. Rev. Genet..

[111] Buenrostro J.D., Wu B., Chang H.Y., Greenleaf W.J. (2015). ATAC-seq: A Method for Assaying Chromatin Accessibility Genome-Wide. Curr. Protoc. Mol. Biol..

[112] Shashikant T., Ettensohn C.A. (2019). Genome-wide analysis of chromatin accessibility using ATAC-seq. Methods Cell Biol..

[113] Baek S., Lee I. (2020). Single-cell ATAC sequencing analysis: From data preprocessing to hypothesis generation. Comput. Struct. Biotechnol. J..

[114] Ponnaluri V.K.C., Zhang G., Esteve P.O., Spracklin G., Sian S., Xu S.Y., Benoukraf T., Pradhan S. (2017). NicE-seq: High resolution open chromatin profiling. Genome Biol..

[115] Yan F., Powell D.R., Curtis D.J., Wong N.C. (2020). From reads to insight: A hitchhiker’s guide to ATAC-seq data analysis. Genome Biol..

[116] Ruiz J.L., Ranford-Cartwright L.C., Gomez-Diaz E. (2020). The regulatory genome of the malaria vector Anopheles gambiae: Integrating chromatin accessibility and gene expression. bioRxiv.

[117] Buenrostro J.D., Giresi P.G., Zaba L.C., Chang H.Y., Greenleaf W.J. (2013). Transposition of native chromatin for fast and sensitive epigenomic profiling of open chromatin, DNA-binding proteins and nucleosome position. Nat. Methods.

[118] Park P.J. (2009). ChIP-seq: Advantages and challenges of a maturing technology. Nat. Rev. Genet..

[119] Siegel T.N., Hekstra D.R., Kemp L.E., Figueiredo L.M., Lowell J.E., Fenyo D., Wang X., Dewell S., Cross G.A. (2009). Four histone variants mark the boundaries of polycistronic transcription units in Trypanosoma brucei. Genes Dev..

[120] Ruiz J.L., Yerbanga R.S., Lefevre T., Ouedraogo J.B., Corces V.G., Gomez-Diaz E. (2019). Chromatin changes in Anopheles gambiae induced by Plasmodium falciparum infection. Epigenet. Chromatin.

[121] Gomez-Diaz E., Rivero A., Chandre F., Corces V.G. (2014). Insights into the epigenomic landscape of the human malaria vector Anopheles gambiae. Front. Genet..

[122] Lukyanchikova V., Nuriddinov M., Belokopytova P., Liang J., Reijnders M.J.M.F., Ruzzante L., Waterhouse R.M., Tu Z., Sharakhov I.V., Fishman V. (2020). Anopheles mosquitoes revealed new principles of 3D genome organization in insects. bioRxiv.

[123] Sim C., Kang D.S., Kim S., Bai X., Denlinger D.L. (2015). Identification of FOXO targets that generate diverse features of the diapause phenotype in the mosquito Culex pipiens. Proc. Natl. Acad. Sci. USA.

[124] Boyle A.P., Davis S., Shulha H.P., Meltzer P., Margulies E.H., Weng Z., Furey T.S., Crawford G.E. (2008). High-resolution mapping and characterization of open chromatin across the genome. Cell.

[125] Galas D.J., Schmitz A. (1978). DNAse footprinting: A simple method for the detection of protein-DNA binding specificity. Nucleic Acids Res..

[126] Tsompana M., Buck M.J. (2014). Chromatin accessibility: A window into the genome. Epigenet. Chromatin.

[127] Song L., Crawford G.E. (2010). DNase-seq: A high-resolution technique for mapping active gene regulatory elements across the genome from mammalian cells. Cold Spring Harb. Protoc..

[128] Pham D.Q., Shaffer J.J., Chavez C.A., Douglass P.L. (2003). Identification and mapping of the promoter for the gene encoding the ferritin heavy-chain homologue of the yellow fever mosquito Aedes aegypti. Insect Biochem. Mol. Biol..

[129] Simon J.M., Giresi P.G., Davis I.J., Lieb J.D. (2012). Using formaldehyde-assisted isolation of regulatory elements (FAIRE) to isolate active regulatory DNA. Nat. Protoc..

[130] Giresi P.G., Lieb J.D. (2009). Isolation of active regulatory elements from eukaryotic chromatin using FAIRE (Formaldehyde Assisted Isolation of Regulatory Elements). Methods.

[131] Behura S.K., Sarro J., Li P., Mysore K., Severson D.W., Emrich S.J., Duman-Scheel M. (2016). High-throughput cis-regulatory element discovery in the vector mosquito Aedes aegypti. BMC Genom..

[132] Perez-Zamorano B., Rosas-Madrigal S., Lozano O.A.M., Castillo Mendez M., Valverde-Garduno V. (2017). Identification of cis-regulatory sequences reveals potential participation of lola and Deaf1 transcription factors in Anopheles gambiae innate immune response. PLoS ONE.

[133] Axel R. (1975). Cleavage of DNA in nuclei and chromatin with staphylococcal nuclease. Biochemistry.

[134] Kuan P.F., Huebert D., Gasch A., Keles S. (2009). A non-homogeneous hidden-state model on first order differences for automatic detection of nucleosome positions. Stat. Appl. Genet. Mol. Biol..

[135] Mieczkowski J., Cook A., Bowman S.K., Mueller B., Alver B.H., Kundu S., Deaton A.M., Urban J.A., Larschan E., Park P.J. (2016). MNase titration reveals differences between nucleosome occupancy and chromatin accessibility. Nat. Commun..

[136] Kensche P.R., Hoeijmakers W.A., Toenhake C.G., Bras M., Chappell L., Berriman M., Bartfai R. (2016). The nucleosome landscape of Plasmodium falciparum reveals chromatin architecture and dynamics of regulatory sequences. Nucleic Acids Res..

[137] Ren Y., Vera D.L., Hughes K.A., Dennis J.H. (2015). Stimulation of the Drosophila immune system alters genome-wide nucleosome occupancy. Genom. Data.

[138] Lay F.D., Kelly T.K., Jones P.A. (2018). Nucleosome Occupancy and Methylome Sequencing (NOMe-seq). Methods Mol. Biol..

[139] Rhie S.K., Schreiner S., Farnham P.J. (2018). Defining Regulatory Elements in the Human Genome Using Nucleosome Occupancy and Methylome Sequencing (NOMe-Seq). Methods Mol. Biol..

[140] Barski A., Cuddapah S., Cui K., Roh T.Y., Schones D.E., Wang Z., Wei G., Chepelev I., Zhao K. (2007). High-resolution profiling of histone methylations in the human genome. Cell.

[141] Han H., Cortez C.C., Yang X., Nichols P.W., Jones P.A., Liang G. (2011). DNA methylation directly silences genes with non-CpG island promoters and establishes a nucleosome occupied promoter. Hum. Mol. Genet..

[142] Natoli G., Andrau J.C. (2012). Noncoding transcription at enhancers: General principles and functional models. Annu. Rev. Genet..

[143] Lam M.T., Li W., Rosenfeld M.G., Glass C.K. (2014). Enhancer RNAs and regulated transcriptional programs. Trends Biochem. Sci..

[144] Mikhaylichenko O., Bondarenko V., Harnett D., Schor I.E., Males M., Viales R.R., Furlong E.E.M. (2018). The degree of enhancer or promoter activity is reflected by the levels and directionality of eRNA transcription. Genes Dev..

[145] Sartorelli V., Lauberth S.M. (2020). Enhancer RNAs are an important regulatory layer of the epigenome. Nat. Struct. Mol. Biol..

[146] Small S., Arnosti D.N. (2020). Transcriptional Enhancers in Drosophila. Genetics.

[147] Miles A., The Anopheles Gambiae 1000 Genomes Consortium, Data Analysis Group (2017). Genetic diversity of the African malaria vector Anopheles gambiae. Nature.

